# LGBTQ-RELATED NEGATIVE LIFE EVENTS AND COGNITION: THE ROLE OF SLEEP, MENTAL DISTRESS AND UNSUPPORTIVE FAMILY

**DOI:** 10.1093/geroni/igae098.4316

**Published:** 2024-12-31

**Authors:** Tara McKay, Anyah Prasad

**Affiliations:** Vanderbilt University, Nashville, Tennessee, United States; Vanderbilt University, Nashville, Tennessee, United States

## Abstract

LGBTQ+ older adults are at increased risk of poor cognition. Using data from the LGBTQ+ Social Networks, Aging and Policy Study (QSNAPS, N=1,202), a unique panel study of 50-76 year old LGBTQ+ adults, and employing Structural Equation Modelling (SEM), we examine the association of LGBTQ-related negative life events (NLEs) with cognitive difficulty. We test whether the effect of NLEs on cognition is mediated by sleep quality or mental distress and whether it is exacerbated by the presence of unsupportive family members. About 20% of respondents report past year experiences of discrimination, harassment and/or violence and 40% report any cognitive difficulty. The association of NLEs with cognitive difficulty (e.g., trouble remembering names) is fully mediated by trouble falling asleep and mental distress (OR=1.21); NLEs increased trouble falling asleep (OR=1.96, p< 0.000), poorer sleep increased the likelihood of severe mental distress (OR=1.32, p< 0.000) and cognitive difficulty (OR=1.09, p<.001), and mental distress increased the odds of cognitive difficulty (OR=2.06, p< 0.001). The presence of unsupportive family members moderates the effect of NLEs on mental distress (main effect: OR=1.15, p=0.67; interaction effect: OR=2.86, p=0.009). The mediation effect of mental distress for the association between NLEs and cognitive difficulty for those with unsupportive family (OR=2.36) is greater than for those with no unsupportive family (OR=1.11). LGBTQ+ adults, especially those with unsupportive family, are at increased risk of cognitive impairment in later life. Interventions to support mental wellbeing, sleep hygiene, and affirmation of LGBTQ+ family members across the life course are critical to achieving health equity in aging.

